# Effects of Huang-Lian-Jie-Du decoction on improving skin barrier function and modulating T helper cell differentiation in 1-chloro-2,4-dinitrobenzene-induced atopic dermatitis mice

**DOI:** 10.3389/fphar.2024.1487402

**Published:** 2024-11-20

**Authors:** Huiyuan Zhang, Quanbin Li, Yaxing Li, Jianhua Guan, Kaidi Li, Yunlong Chen

**Affiliations:** ^1^ School of Chinese Materia Medica, Tianjin University of Traditional Chinese Medicine, Tianjin, China; ^2^ Hubei College of Chinese Medicine, Jing Zhou, Hubei Province, China; ^3^ School of Medical Technology, Tianjin University of Traditional Chinese Medicine, Tianjin, China; ^4^ Tianjin Key Laboratory of Therapeutic Substance of Traditional Chinese Medicine, Tianjin University of Traditional Chinese Medicine, Tianjin, China

**Keywords:** atopic dermatitis, Huang-Lian-Jie-Du decoction, skin barrier, OX40, OX40L, T helper cells

## Abstract

**Background:** Atopic dermatitis (AD) is among the most frequently encountered skin diseases, bothering a considerable number of patients. Today, corticosteroids and antihistamines are among the numerous drugs applied for the therapy of AD. However, lengthy use of them contributes to side effects, such as physiological changes in skin. As an alternative and supplementary therapy, traditional Chinese medicine has become a trend for AD treatment. Huang-Lian-Jie-Du decoction (HLJDD), a renowned herbal formula has been employed to treat inflammatory diseases such as AD. However, its role in regulating immunity in AD remains unclear. The object of this study was to elucidate the efficacy of HLJDD and reveal the implicit mechanism from an immunological perspective in AD-like mice.

**Methods:** In brief, 1-chloro-2,4-dinitrobenzene (DNCB) for the sensitization phase (1% DNCB) and stimulation phase (1.5% DNCB) were applied for BALB/c mice. HLJDD and dexamethasone (DXMS) were administered orally to the mice. Mice skin and spleens were collected to evaluate the efficacy of HLJDD. 16S rRNA sequencing was applied to evaluate the commensal microbiota changes in skin and fecal. *In vitro*, spleen CD4^+^ T cells and bone marrow-derived mast cells (BMMCs) were co-cultured to explore the modulation of HLJDD in T helper (Th) cells phenotyping.

**Results:** HLJDD showcased a substantial amelioration in skin through the upregulation of FLG, LOR, AQP3, and reducing scratching behaviors in AD-like mice, Also, the quantity of infiltrated mast cells (MCs), pruritus-related mRNA were decreased. In addition, the expression of OX40/OX40L was decreased by HLJDD, which was critical in Th-cell phenotyping. With the treatment of HLJDD, Th1/Th2 and Th17/Treg ratios in AD-like mice became balanced. The structure of commensal microbiota in AD-like mice was affected by HLJDD. HLJDD could also improve the imbalance of Th17/Treg *in vitro*.

**Conclusion:** HLJDD could improve the symptoms of AD-like mice by alleviating the scratching behaviors via decreased Th2 and pruritus-related mRNA expression. HLJDD also enhanced the relative diversity of skin microbiota and changed the structure of intestinal microbiota. An in-depth study found that HLJDD could balance the ratio of Th1/Th2, Th17/Treg in AD-like mice, and Th17/Treg *in vitro* by regulating the OX40/OX40L signaling pathway.

## 1 Introduction

Atopic dermatitis (AD) characterized by dry, itchy, erythematous skin, is a recurrent, chronic skin disease (Sroka-Tomaszewska and Trzeciak, 2021). The global prevalence of AD is on the increase, 60% of AD patients are infants, and 10% of adults suffer from AD. (Langan et al., 2020; Sacotte and Silverberg, 2018; Weidinger and Novak, 2016). Patients and their families bear enormous physical, psychological, and economic burdens from AD (Ständer, 2021).

Skin-dysregulated immunity and barrier dysfunction are risky factors in the development of AD (Yang et al., 2020). Generally, damaged skin barrier function brings about skin inflammation and chronic itch (Tominaga and Takamori, 2022). The itching sensation has been linked to both epithelial and immune cells, which are thought to play a role in triggering the itch-scratch cycle. This, in turn, has the potential to contribute to the development of malignant behaviour through repeated scratching (Mack and Kim, 2018). The itch-scratch cycle further disrupts the skin barrier, prompting keratinocytes to release pruritogenic cytokines such as interleukin-31 (IL-31) (Barbarot, 2022). Immune dysregulation in AD is associated with an imbalance in T helper 1 (Th1), Th2, and Th17, Treg cell-associated immune responses (Ma et al., 2014; Sheikhi et al., 2017). Th2-mediated immune response releases IL-4 and IL-13 that damage skin barrier function, induce itching, and stimulate the production of additional inflammatory factors (Steinhoff et al., 2022). Compromised skin barriers are more susceptible to allergens, and increase the risk for atopic March such as food allergies and asthma (Wang et al., 2021). Currently, the therapy of AD aims to alleviate symptoms and reduce environmental factors to minimize relapse (Guo et al., 2023). Topical anti-inflammatory therapy, includes tacrolimus, phosphodiesterase 4 inhibitor crisaborole, as well as topical corticosteroids (TCS). However, lengthy use of TCS could cause skin telangiectasia and atrophy (Kleinman, et al., 2022). Antipruritic therapy, targeted biological therapies, and phototherapy are effective treatments for AD (Li et al., 2021). Nevertheless, these approaches may result in heavy financial burdens for AD patients (Gatmaitan and Lee, 2023). Relieving inflammation with medicinal plants is proposed as a substitute method for conventional treatments (Tasneem et al., 2019). Thus, finding a safe and cost-effective treatment that could regulate the immune system and alleviate the skin symptoms of AD is imperative.

Huang-Lian-Jie-Du decoction (HLJDD) is a traditional Chinese herbal formula, which consists of four medicinal plants: *Coptis chinensis* Franch. (Huang-Lian, Coptidis Rhizoma), *Scutellaria baicalensis* Georgi (Huang-Qin, Scutellariae Radix), the bark of *Phellodendron chinense* C. K. Schneid. (Huang-Bai, Phellodendri Chinensis Cortex), and the fruit of *Gardenia jasminoide* J. Ellis (Zhi-Zi, Gardenia Fructus), in a ratio of 3:2:2:3. It was initially documented in Wang Tao’s treatise, “Wai Tai Mi Yao” in the Tang Dynasty (Qi et al., 2019). In clinical practice, HLJDD and its modified formulas have demonstrated efficacy in the treatment of patients with AD and psoriasis. The most prevalent method of administration is a decoction of water, which is taken orally (Suqing and Yuepeng, 2017). In our previous studies, the evidence we found that HLJDD could suppress the generation of inflammatory mediators in stimulated RAW264.7 cells and RBL-2H3 cells, and alleviate skin lesions in AD-like mice (Chen et al., 2016; Chen et al., 2020). Nevertheless, it is unclear how HLJDD regulates immunity and reduces pruritus. In the current research, it is intended to explore the implicit mechanisms of HLJDD in regulating immunity and relieving inflammation in 1-chloro-2,4-dinitrobenzene (DNCB)-induced AD-like mice.

## 2 Materials and methods

### 2.1 Reagents and antibodies

The dispensing granules of traditional Chinese medicines (TCMs) is an innovative preparation of medicinal material for TCMs decoction. And the granules of HLJDD were sourced from Baokang Affiliated Hospital of Tianjin University of Traditional Chinese Medicine, and subsequently utilized in this study (Batch number: 22032100171). The composition of HLJDD was shown in Table 1. 10.3g of the dispensing granules of HLJDD were equivalent to the raw medicinal herbs of Coptis chinensis Franch. (9      g), Scutellaria baicalensis Georgi (6g), Phellodendron chinense Schneid. (6g), Gardenia jasminoide J. Ellis (9g). Dexamethasone (DXMS, D137736, Aladdin), 1-chloro-2,4-dini-trobenzene (DNCB, purity ≥99%, TCI, 97–007), acetone (2,853, Damao), methanol (A452-4, Thermo Fisher), olive oil (8,001–25-0, Aladdin), berberine, phellodendrine, geniposide, baicalin (DX0009, DH0031, DZ0032, DH0023, LEMEITIAN MEDICINE). Hematoxylin-Eosin Stain Kit, Toluidine Blue O Solution (H&E, TB, G1120, G3662, Solarbio). Recombinant-murine-SCF and recombinant-murine-IL-3 (250–03, 213–13, Peprotech). DNP-HSA, Anti-DNP-IgE, PMA, Ionomycin (A6661, D8406, P8139, I3909, Sigma-Aldrich), Golgistop (554,724, BD). The primary antibodies applied for protein detection including OX40, β-Actin and HRP-conjugated secondary Antibodies (61637S, 4970S, 7074S, CST), OX40L Antibody (MAB1236, R&D), LOR Antibody (PA530583, Thermo Fisher), AQP3 Antibody (ab125219, Abcam), Filaggrin Antibody (66,192, Santa Cruz). PageRuler (26,616, Thermo Fisher)Anti-mouse CD3ε, anti-mouse CD28, CD16/32, anti-OX40L, FITC-CD3, PerCP-Cy5.5-CD4, APC-IFN-γ, PE-IL-4, APC-CD25, APC-IL-17A, PE-FOXP3, APC-FεRIα, FITC-CD117 Antibodies (100,340, 102,116, 156,603, 108,802, 100,203, 100,433, 505,809, 504,103, 506,915, 101,910, 126,403, 134,315, 161,603, Biolegend), Cell Counting Kit-8 (CCK-8, C0038, Beyotime), SYBR Green (FP205, TINGEN), RPMI-1640 medium (A1049101, Gbico).

**TABLE 1:** Component information in HLJDD.

Full botanical plant names	Pharmaceutical name	Chinese name	Part
*Coptis chinensis* Franch	Coptidis Rhizoma	Huang-Lian	Rhizome
*Scutellaria baicalensis* Georgi	Scutellariae Radix	Huang-Qin	Rhizome
*Phellodendron chinense* C. K. Schneid	Phellodendri Chinensis Cortex	Huang-Bai	Bark
*Gardenia jasminoide* J.Ellis	Gardenia Fructus	Zhi-Zi	Fruit

### 2.2 Quantitative analysis of HLJDD

1 mL of methanol was added into 5 mg of HLJDD, sonicated for 30 min, and centrifuge the extract (12000g, 4°C, 15 min); The supernatant was filted through a 0.22 μm membrane. 10 μL of the subsequent filtrate was injected into the high performance liquid chromatography (HPLC, Alliance e2695, COSMOSIL 5C_18_-MS-II 4.6 mm I.D. × 250 mm). The mobile phase consisted of a 0.1% formic acid aqueous solution (A) - methanol (B), column temperature: 30°C, detection wavelength: 238 nm, flow rate: 1 mL/min, injection volume 20 μL, gradient elution profile was set as follows: 0–10 min, 10% B; 10–12 min, 10%–15% B; 12–14 min, 15%–20% B; 14–16 min, 20%–30% B; 16–17 min, 30%–31% B; 17–21 min, 31%–31.4% B; 21–30 min, 31.4%–35% B; 30–35 min, 35%–47% B; 35–40 min, 47%–50% B; 40–50 min, 50%–65% B.

### 2.3 Mice and treatment

BALB/c mice (female, six-to eight-week-old) were purchased from Beijing Sibei Fu Biotechnology Company. Animals were maintained on a 12-h light/dark cycle under temperature (23°C ± 1°C) and provided with standard diet and water *ad libitum*. Animals were administered by skilled personnel in a specific pathogen free environment at the Animal Research Center of Tianjin University of Traditional Chinese Medicine. The mice were provided *ad libitum* access to diet and water (TCM-LAEC2022098).

### 2.4 Induction and treatment of AD-like skin lesions in mice

One week following acclimatization, mice were randomly allocated into six groups with ten mice of each: (1) Control group; (2) Model group (DNCB); (3) DXMS group (DNCB+2.5 mg/kg DXMS); (4) Low dose of HLJDD group (DNCB +400 mg/kg HLJDD); (5) Medium dose of HLJDD group (DNCB +800 mg/kg HLJDD); (6) High dose of HLJDD group (DNCB+1600 mg/kg HLJDD). The mice were administered DXMS, HLJDD, or normal saline orally in the elicitation phase. The experimental induction of AD-like mice in this study utilized an established murine model with slight modifications (Chen et al., 2020). The procedure is depicted in Figure 2A. In brief, the dorsal hair of mice was shaved under anesthesia. Subsequently, 200 μL of blank solution (acetone/olive oil, 3:1, v/v) or 1% DNCB (DNCB dissolved in blank solution) was utilized on the shaved skin on days 1, 2 and 3. The mice were subjected to challenge with 200 μL of 1.5% DNCB or blank solution on the same dorsal skin on day 14, 17, 20, 23, 26. The mice were sacrificed on day 29, the skin and spleen samples were collected for the further analysis.

### 2.5 Dermatitis score

The AD-like dermatitis severity was assessed on the day preceding sacrifice: (1) erythema, (2) oozing/crusting, (3) dryness/scaling, and (4) excoriation/erosion. The four individual symptom scores ranged from 0 to 3. (0, no symptom; 1, mild; 2, moderate; 3, severe). The final dermatitis severity score consisted of the sum of these scores.

### 2.6 Measurement of Transepidermal Water Loss and scratching behavior

Transepidermal Water Loss (TEWL) values were measured before sacrifice by VapoMeter (SWL5201, Delfin). Behaviors of scratching were recorded using a digital camera for a duration of 20 min before the endpoint of the experiment.

### 2.7 Histopathological analysis

In assessing the skin lesions and inflammation, lesional skin were adequately fixed in 4% paraformaldehyde for subsequent paraffin-embedded skin sections. Then, H&E staining were applied to assess the epidermal thickness and skin pathological changes. Additionally, infiltration of mast cells (MCs) was identified through TB staining. The picture of the skin section was taken by EVOS M7000 (Invitrogen). The epidermal thickness and the infiltrated MCs were randomly selected in three areas in each section to calculate the mean value.

### 2.8 Western blot

Protein samples were extracted from lesional skin and quantitated. Then, proteins were loaded onto a 10% gradient sodium dodecyl sulfate-polyacrylamide gel electrophoresis (SDS-PAGE) and transferred to a PVDF membrane. Then, membranes were blocked with 5% nonfat milk. The membranes were incubated overnight at 4°C with primary antibodies specific to loricrin (LOR), aquaporin 3 (AQP3), OX40, OX40L, or β-actin. Finally, secondary antibodies were incubated with membranes. All protein bands in the PVDF membrane were assessed utilising the ChemiDoc MP Imaging system and quantified by ImageJ.

### 2.9 Pruritogenic-related factors of mRNA expression by RT-qPCR

The RNA was extracted using the Silica Column Method (Promega), and RNA was reverse transcribed to complementary DNA by reverse transcription kit (KR118, TINGEN). Relative expression of thymic stromal lymphopoietin (TSLP), interleukin-13 (IL-13), histamine H4 receptor (HRH4), interleukin-31 (IL-31), and GAPDH were assessed by using SYBR Green, normalized to the expression of GAPDH. The primer (Sangon Biotech) sets utilized were as shown in Table 2. In this study, the 2^−ΔΔCT^ method was employed to quantify the relative mRNA expression, with β-actin serving as the normalised reference.

**TABLE 2:** Primers used in RT-qPCR.

Gene	F/R	Sequence (5'-3′)
TSLP	F	GAG​CAA​ATC​GAG​GAC​TGT​GAG​AGC
	R	AGT​GAA​GGG​CAG​CCA​GGG​ATA
IL-13	F	CTC​TTG​CTT​GCC​TTG​GTG​GTC​TC
	R	GGG​AGT​CTG​GTC​TTG​TGT​GAT​GTT​G
IL-31	F	CAC​AGG​AAC​AAC​GAA​GCC​TAC​CC
	R	GAT​ATT​GGG​GCA​CCG​AAG​GAC​AAG
HRH4	F	GAA​GAA​CAG​GAA​CAC​AAA​GGA​C
	R	AGT​GAA​GGG​CAG​CCA​GGG​ATA​G
GAPDH	F	ATG​GGG​AAG​GTG​AAG​GTC​G
	R	GGG​GTC​ATT​GAT​GGC​AAC​AAT​A

### 2.10 Immunohistochemistry for skin paraffin-embedded sections

In accordance with the manufacturer’s guidelines (SA1021, BOSTER), the endogenous peroxidase was neutralized following the deparaffinization and rehydration of the tissue sections. Subsequently, the tissue was permeabilized for 10 min with Triton X-100. The sections were placed in citrate antigen retrieval solution and heated to 95°C for 20 min. Afterwards, the samples were cooled to room temperature and incubated with FLG and secondary antibodies, respectively. The cell nuclei were stained with hematoxylin, sealed, and photographed under a microscope. ImageJ was employed to calculate the positive area of each field of view.

### 2.11 16S rRNA gene sequencing

In summary, microbial genomic DNA was extracted from each skin and fecal sample, and the V3 and V4 hypervariable regions of the 16S rRNA gene were amplified. The library was sequenced on Illumina Novaseq 6,000. The abundance and diversity of microbiota in mice were measured using the PacBio sequencing platform (Biomarker Technologies, Beijing, China).

### 2.12 Isolation and identification of bone marrow-derived mast cells and naive CD4^+^T cells

Bone marrow-derived mast cells (BMMCs) were isolated from the femora of male C57BL/6 mice aged 6–8 weeks. Then, the bone marrow cells were cultured in RPMI-1640 medium containing 10% FBS, 1% P/S, and supplemented with murine IL-3、SCF (10 ng/mL). Following 4–6 weeks of cell induction, the mast cell phenotype was characterized by the expression of FεRIα and CD117 assessed via flow cytometry using APC-FεRIα antibody and FITC-CD117 antibody.

Spleens of C57BL/6 mice were applied to isolate naive CD4^+^ T cells using the CD4 naive T Cell isolation Kit and Magnet (480,040, 480,020, Biolegend). In brief, the purity of CD3^+^CD4^+^ T cells was identified by FITC- CD3 and PerCP-Cy5.5-CD4 antibodies via flow cytometry.

### 2.13 BMMCs and T cell viability assay

CCK-8 assay was conducted to assess T cells and mast cells viability. Briefly, 10^4^ cells/well were incubated in a 96-well plate with HLJDD (0, 1.5625, 3.125, 6.25, 12.5, 25, 50 μg/mL). CCK-8 solution (10 μL per well) was added, and the 96-well plate was then incubated for 2 h. The absorbance was measured at 450 nm.

### 2.14 BMMCs-T cell coculture

BMMCs were treated with 5 μg/mL CD16/32 antibody, then sensitized with anti-DNP IgE (1 μg/mL) overnight. Naive CD4^+^ T cells were incubated in a 6-well plate coated with anti-mouse CD3ε antibody (5 μg/mL) and supplemented with anti-mouse CD28 antibody (5 μg/mL) overnight. The sensitized BMMCs and T cells were cultured in RPMI 1640 medium at a 1:1 ratio. Before challenging, the Anti-OX40L antibody was added as the blocker group, and the HLJDD group was pretreated with different doses of HLJDD for 1 h. Finally, all groups were challenged with 100 ng/mL DNP-HSA. The BMMCs of the control group were absent of anti-DNP IgE sensitization and DNP-HSA challenge.

### 2.15 Flow cytometric analysis *in vivo* and *in vitro* studies

Spleen cells were isolated and stimulated with PMA (50 ng/mL), Inomycin (1 μg/mL) and Golgistop (4 μL) for 5 h. Cells were stained surface marker with PerCP-Cy5.5-CD4 antibody, then stained intracellularly with APC-IFN-γ antibody, PE-IL-4 antibody to detect the rate of Th1/Th2 cells. Th17 cells were identified through surface staining with PerCP-Cy5.5-CD4 antibody and APC-IL-17A antibody for Th17 cells. Tregs were characterized via surface staining with PerCP-Cy5.5 CD4 antibody and APC-CD25 antibody. Finally, intracellular staining with PE-FOXP3 antibody.

To investigate the impact of HLJDD on Th1/Th2 and Th17/Treg shifts *in vitro*, naive CD4^+^ T cells were isolated from C57BL/6 spleens and stimulated, stained, and detected as described above, and data were analyzed using FlowJo.

### 2.16 Statistical analysis

The data obtained from the experiment were statistically processed by GraphPad Prism (version 9.0.0), and expressed as mean ± SEM. In parametric data, one-way analysis of variance (ANOVA) (Dunnett’s and Tukey’s multiple comparison test) was used for comparing data across multiple-group comparisons. The non-parametric test was employed for the analysis of RT-qPCR and microbiota data. A significance threshold of *p* < 0.05 threshold was utilized to determine statistical significance.

## 3 Results

### 3.1 Quantitative analysis of HLJDD by HPLC

**FIGURE 1:**
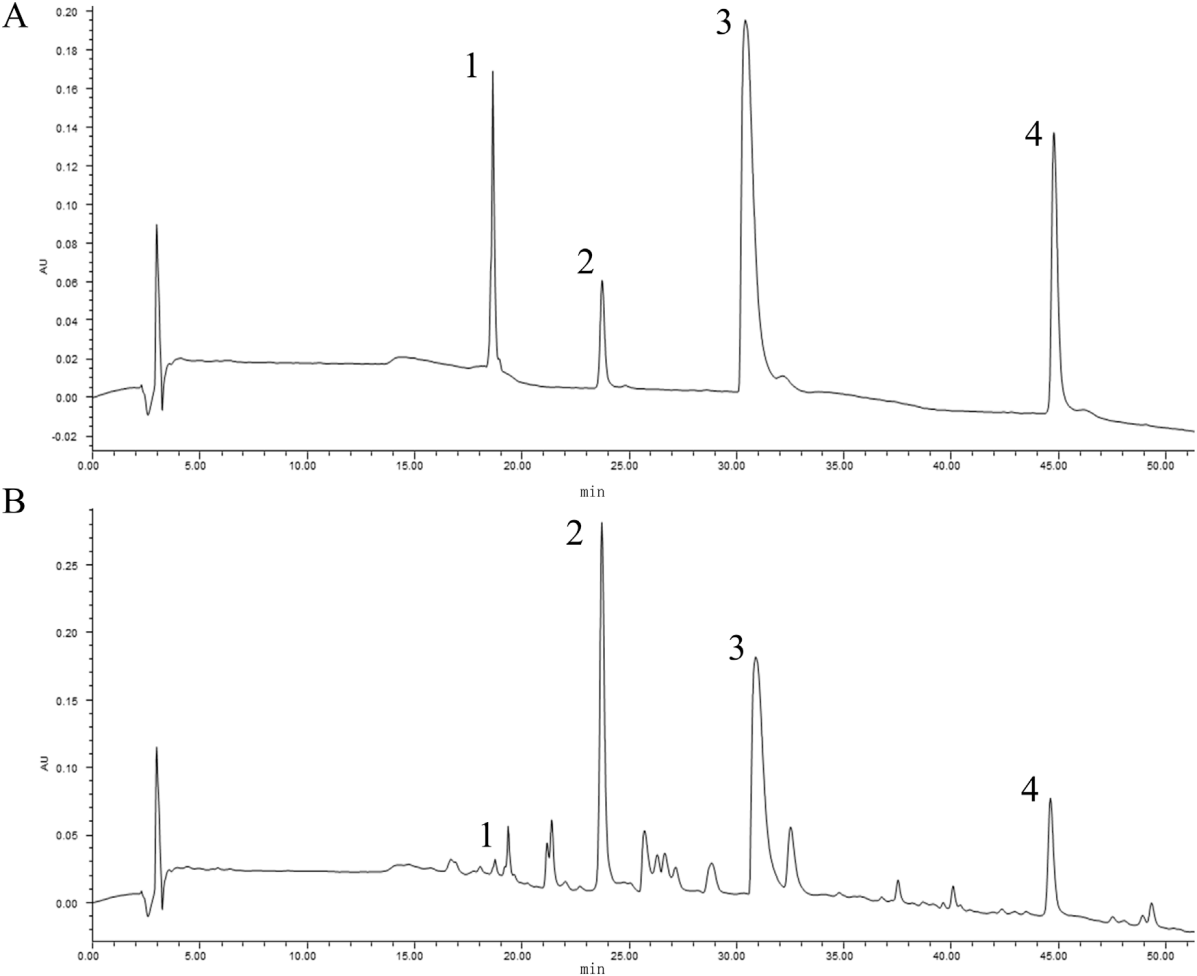
Quantitative analysis of HLJDD by HPLC. The peaks indicated by the black numbers in the figure represent the identified metabolites, phellodendrine (1), geniposide (2), berberine (3), baicalin (4). The HPLC chromatogram of mixed references **(A)** and the chromatogram of HLJDD **(B)**.

In accordance with the National Medical Products Standards, the principal metabolite of Coptidis Rhizoma granules is berberine. Similarly, baicalin represents the primary metabolite of Scutellariae Radix granules, phellodendrine is the predominant metabolite of Phellodendri Chinensis Cortex granules, and geniposide is the principal metabolite of Gardenia Fructus granules. The contents of the four major metabolites in HLJDD were determined to be 0.61% (phellodendrine), 6.48% (geniposide), 3.87% (berberine), and 3.84% (baicalin), based on the calculation of pre-constructed standard curves (Figure 1).

### 3.2 Amelioration of skin lesion symptoms in AD-like mice by HLJDD

**FIGURE 2:**
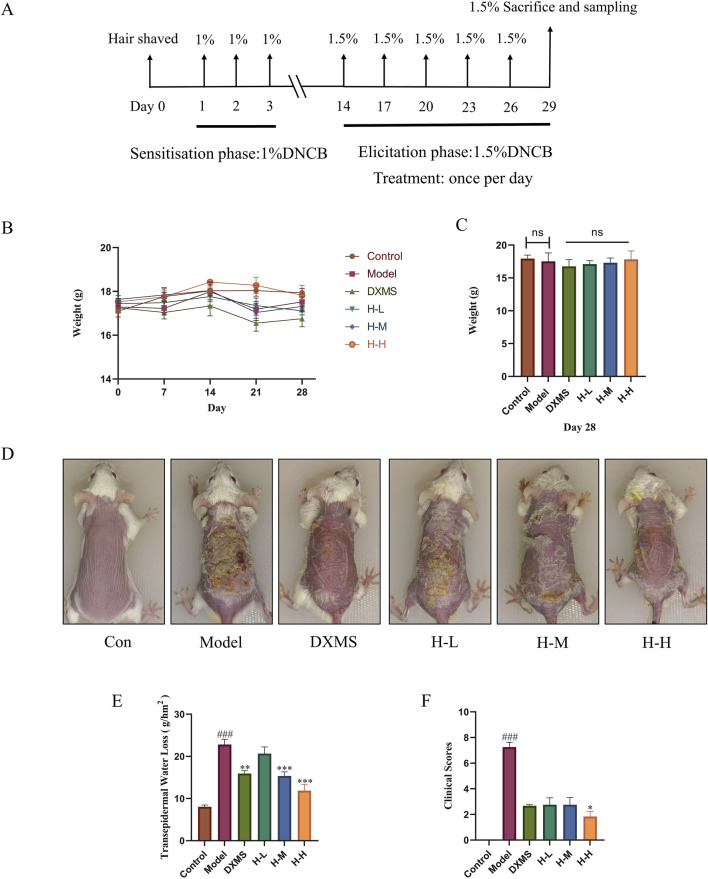
The administration of HLJDD alleviated the clinical symptoms of AD-like mice. Experimental protocol for AD-like mice **(A)** From day 1 to day 3, 1% DNCB solution was applied on the dorsal skin and then challenged with 1.5% DNCB solution every 3 days. **(B, C)** Body weight of mice at 0, 7, 14, 21, 28 days, and body weight of mice in each group on day 28 (n = 8) **(D)** Representative photos of mice skin in each group before sacrifice. **(E)** TEWL in each group of mice at the experiment endpoint (n = 6) **(F)** Clinical scores of mice skin at the experiment endpoint (n = 6). Data were the mean ± SEM. Control group (Con), Model group (Model), Dexamethasone group (DXMS), Low, medium, and high doses of Huang-Lian-Jie-Du Decoction (H-L, H-M and (H–H). ###*p* < 0.001 vs control group; *p < 0.05, **p < 0.01, and ***p < 0.001 vs model group.

The procedure of establishing AD-like mice model was depicted in Figure 2A. The body weight of the DXMS group exhibited a slight decline with no statistically significant difference. Furthermore, the administration of HLJDD did not result in a notable alteration in the body weight of the mice (Figures 2B, C). The model group exhibited severe skin lesion symptoms. Compared with the control group, the model group showcased severe epidermal damage such as crusts, erythema, lichenification, and excoriations, with significantly increased clinical scores (Figures 2D, F). TEWL was increased by approximately 3-fold elevation. The TEWL after treatment with HLJDD or DXMS and the skin lesion symptoms in each treatment group were considerably ameliorated compared to the model group. The value of TEWL in HLJDD groups showed a dose-dependently reduction (Figure 2E). This result showed that the HLJDD group could pose a positive effect on clinical scores (Figure 2F). Therefore, HLJDD could markedly ameliorate the symptoms of skin lesions in DNCB-treated mice.

### 3.3 Effects of HLJDD on histological changes in the epidermal tissue of AD-like mice

**FIGURE 3:**
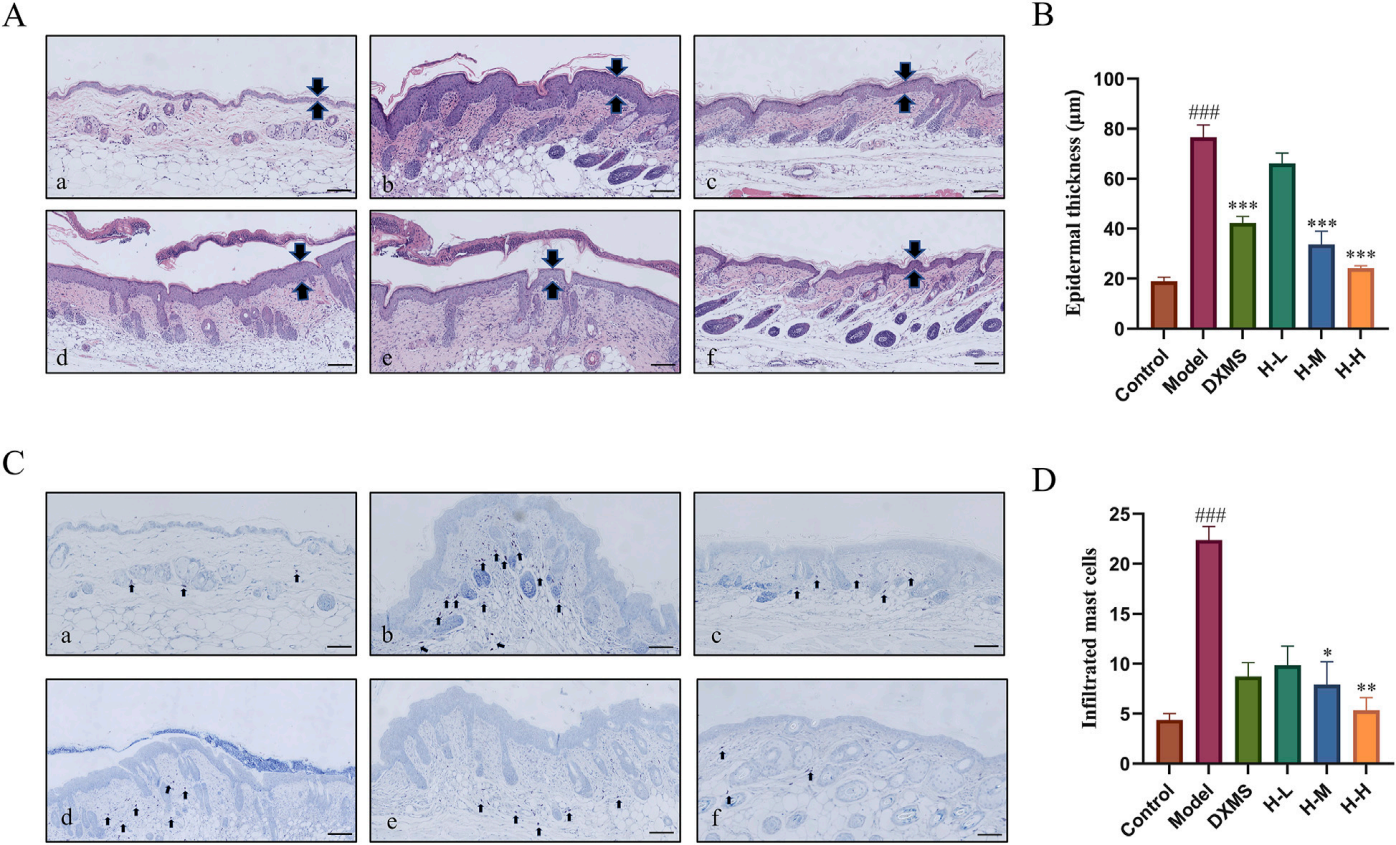
Effects of HLJDD on histological changes in the epidermal tissue of AD-like mice. **(A)** Control group, **(B)** Model group, **(C)** DXMS group, **(D)** H-L group, **(E)** H-M group, **(F)** H-H group **(A–D)** H&E and TB staining of skin sections, and analyzed for epidermal thickness, mast cell infiltration (scale bar = 100 μm, n = 6). Data were the mean ± SEM. ###*p* < 0.001 vs control group; *p < 0.05, **p < 0.01, and ***p < 0.001 vs model group.

The results showed that there was a complete skin structure in the control group. There were 5.11-fold and 4.05-fold increases in the epidermal and infiltrated MCs among AD-like mice. The model group showcased excessive keratinization of the epidermis, thickening of the epidermal, significant sponge-like edema, and a large quantity of inflammatory cells infiltrated into the skin dermis. After treatment with DXMS and HLJDD, the pathological condition of the skin improved (Figure 3A). The H-M and H-H groups showed a markable reduction in the epidermal thickness (Figure 3B).

A large number of MCs was infiltrated in the skin of the model group which was markedly higher than that of the control group (Figure 3C). The treatment groups considerably reduced the amount of MCs infiltration (Figure 3D). It indicated that HLJDD could noticeably improve the pathological changes of skin tissue and ameliorate inflammatory symptoms of skin in AD-like mice.

### 3.4 Effects of HLJDD on itching symptoms and the level of pruritus-related mRNA

**FIGURE 4:**
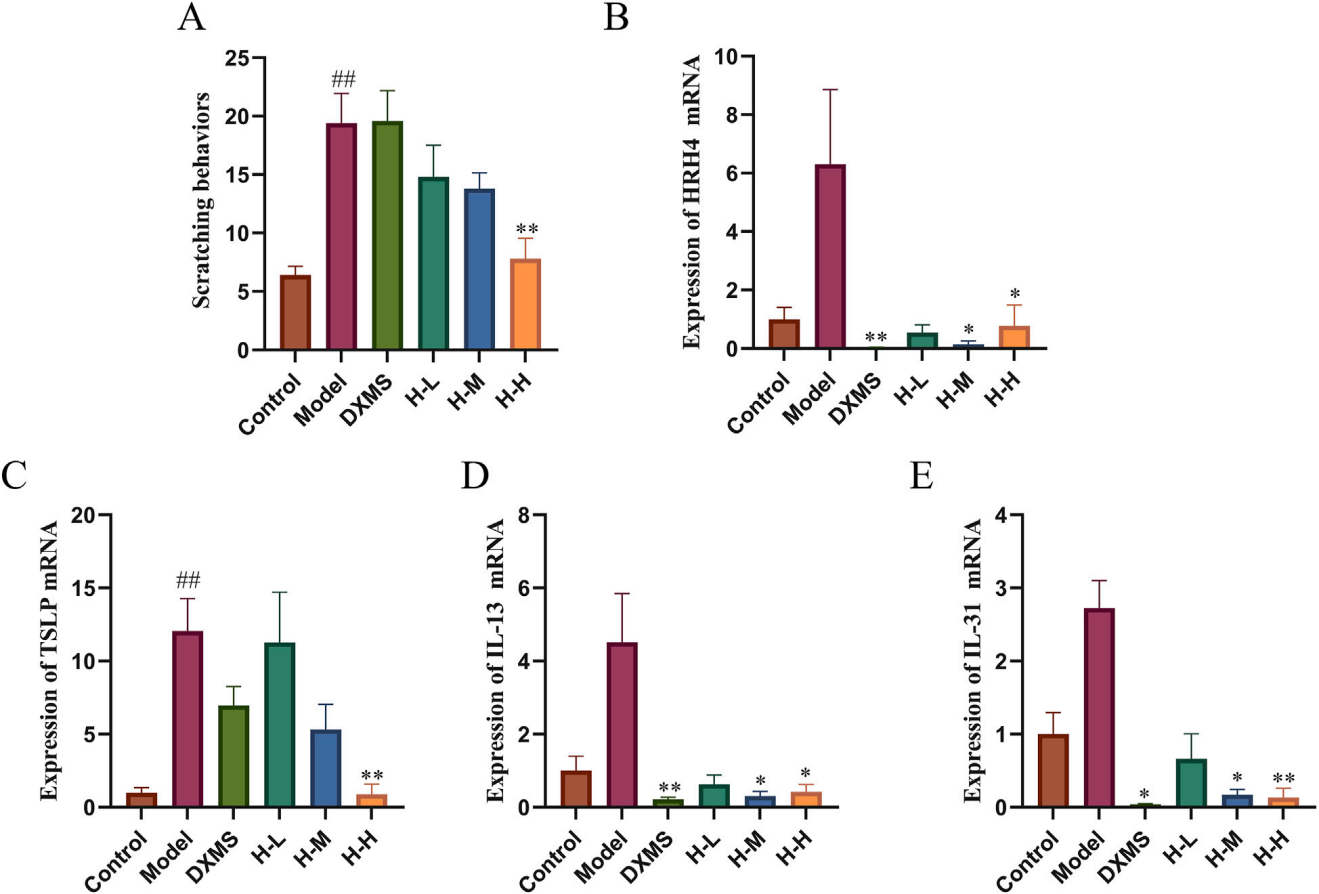
Effects of HLJDD on itching symptoms and mRNA level of HRH4, TSLP, IL-13, and IL-31 **(A)** Number of scratches in 20 minutes (n = 5) **(B–E)** Relative mRNA expression of HRH4, TSLP, IL-13, and IL-31 (n = 3). Data were the mean ± SEM. ##*p* < 0.01 vs control group; *p < 0.05, **p < 0.01 vs model group.

As shown in Figure 4A, a 3.03-fold elevation of scratching behaviors was observed in AD-like mice. Scratching behaviors in the DXMS group did not change significantly compared with the model group. However, HLJDD groups exerted a reduction of scratching behaviors in a dose-dependent manner, which was consistent with the results of TB staining (Figure 3C). The skin tissues in the model group showed an increased expression of HRH4, TSLP, IL-13, and IL-31 mRNA. HRH4, IL-13, and IL-31 mRNA levels were reduced in the epidermal tissue after 2 weeks of treatment with the drug. However, the DXMS group did not significantly reduce the mRNA level of TSLP, which may be why DXMS did not considerably decrease scratching behaviors in mice (Figures 4B–E). HLJDD alleviated itching in AD mice with the reduction of epidermal mast cell infiltration and downregulation of itch-related factors, including IL-13, HRH4, TSLP, and IL-31 mRNA.

### 3.5 Effects of HLJDD on skin barrier restoration and on OX40/OX40L pathway

**FIGURE 5:**
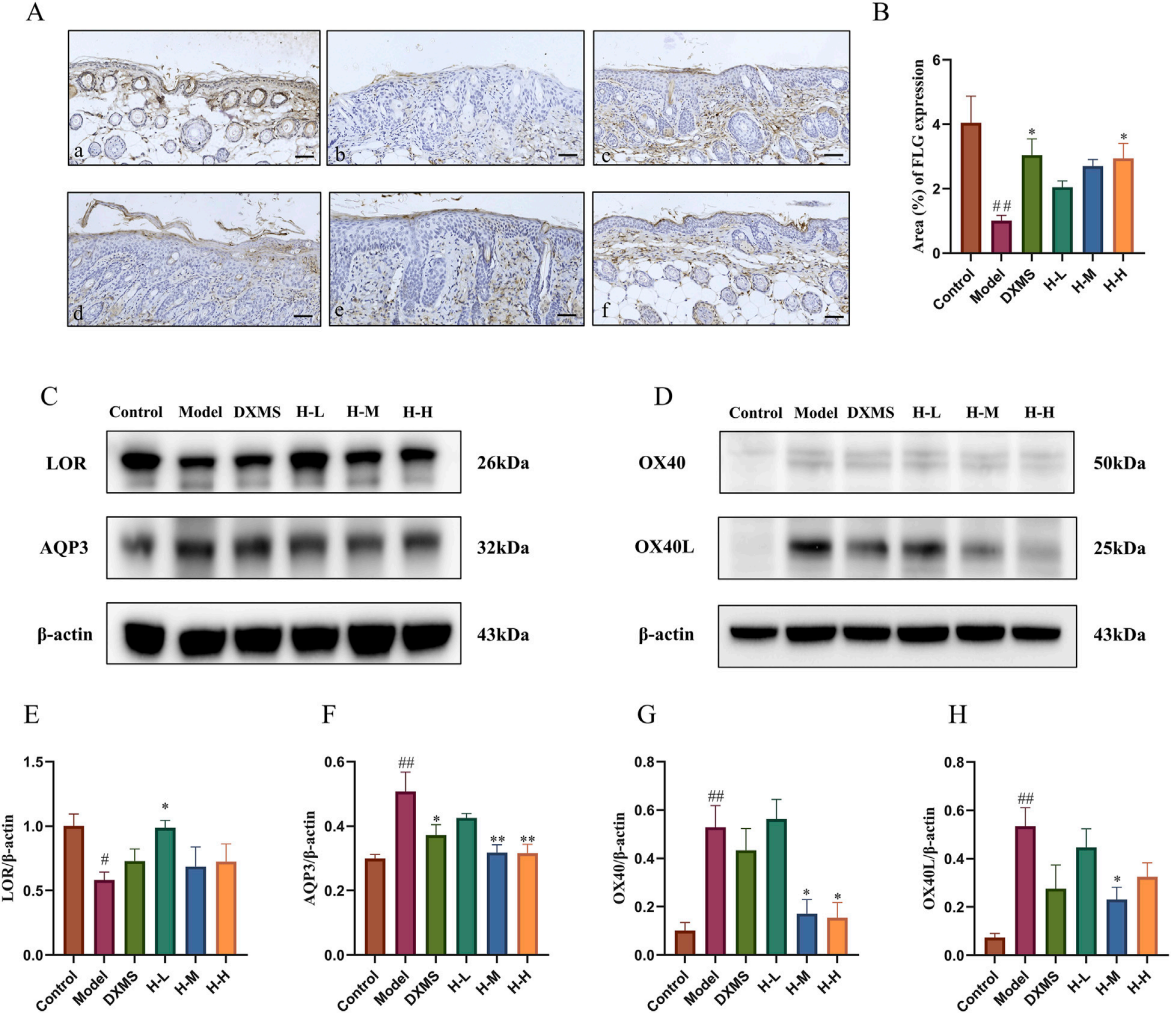
Effects of HLJDD on skin barrier restoration and on OX40/OX40L pathway in AD-like mice **(A, B)** Representative images of immunohistochemistry labelling and quantification of the expression of FLG (scale bar = 100 μm, n = 3) **(C, D)** Representative protein bands on the expression of LOR, AQP3, and OX40, OX40L with β-actin being used as the control (n = 3) **(E–H)** Quantification of LOR, AQP3, OX40, and OX40L bands (n = 3). Data were the mean ± SEM (n = 3). #*p* < 0.05, ##*p* < 0.01 vs control group; *p < 0.05, **p < 0.01 vs model group.

FLG, LOR and AQP3 are both vital in skin barrier function (Bollag et al., 2020; Schmuth et al., 2024). The immunohistochemical results demonstrated a notable suppression of FLG expression in the model group, whereas the H-H group exhibited an increase in FLG expression, which had a favorable impact on the restoration of the skin barrier (Figures 5A, B). AD-like mice showed the decreased expression of LOR and AQP3 on the dorsal skin (Figure 5C), which was consistent with the trend of TEWL (Figure 2E). After treatment with HLJDD, expression of LOR was upregulated in the HLJDD group, especially in the low-dose HLJDD group (Figures 5C, E). H-M and H-H groups showed similar inhibition of the expression of AQP3 (Figures 5C, F). Thus, HLJDD could reduce TEWL by enhancing the epidermal barrier via regulating the expression of FLG, LOR and AQP3 to treat the symptoms of skin lesions in AD-like mice.

Studies have shown that the binding of OX40 and OX40L has an essential effect on T cell differentiation, and the targeting of OX40/OX40L in the treatment of AD is currently a hot research topic (Elieh Ali Komi and Grauwet, 2018). This study showed that the upregulated expression of OX40 and OX40L in AD-like mice was markedly downregulated after treatment of HLJDD (Figures 5D, G, H). The result indicated that the mechanism of HLJDD treating AD may impact the immune response, particularly in T cell differentiation, so we then detected the Th1/Th2 and Th17/Treg ratios in the spleen using flow cytometry.

### 3.6 Effects of HLJDD on the regulation of spleen Th1/Th2 and Th17/Treg cells in AD-like mice

**FIGURE 6:**
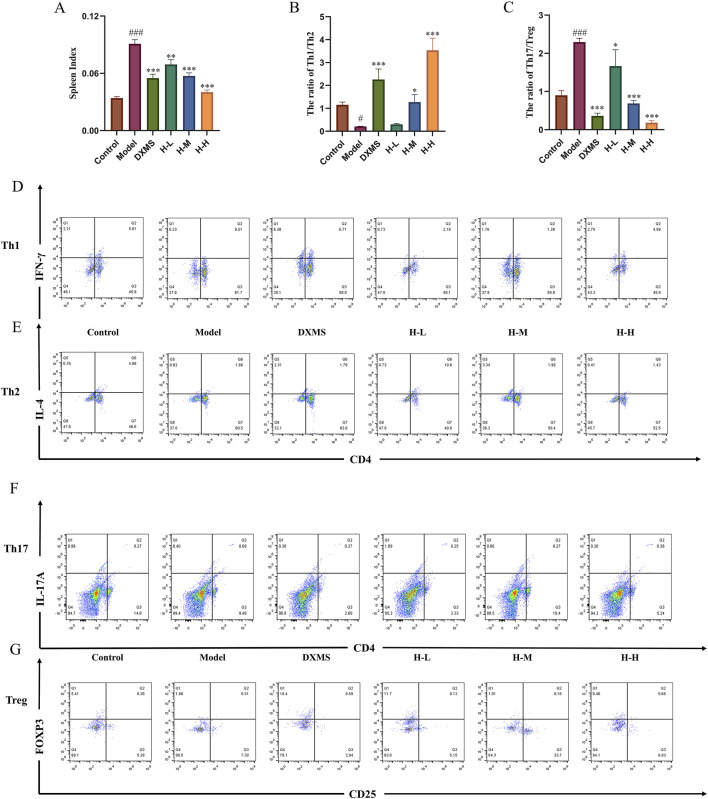
Effects of HLJDD on regulating spleen Th1/Th2 and Th17/Treg in AD-like mice **(A)** Spleen index of mice in each group (n = 6) **(B–C)** Spleen Th1/Th2 and Th17/Treg ratios of mice in each group. **(D–G)** Representative images of Th1/Th2/Th17/Treg detected by flow cytometry (n = 3). Data were the mean ± SEM. #*p* < 0.05, ###*p* < 0.001 vs control group; *p < 0.05, **p < 0.01, and ***p < 0.001 vs model group.

AD patients in the acute phase, a remarkable hyperactivity of Th2 and Th17 cells is observed. (Tokura and Hayano, 2022). T cells constitute a crucial element of the adaptive immune system, exhibiting a pervasive distribution throughout the spleen. (Lewis et al., 2019). The markedly increased spleen index compared with the control group indicated a change in immunity related to T cells (Figure 6A). The results of flow cytometry showed a significant downregulation of Th1/Th2, and upregulation of Th17/Treg ratios in the model group. The treatment with DXMS and HLJDD led to significantly declined spleen index of AD-like mice, and Th1/Th2 and Th17/Treg ratios were changed. Among them, DXMS noticeably upregulated the Th1/Th2 ratio and downregulated the Th17/Treg ratio in the spleen lymphocytes of AD mice. HLJDD could significantly downregulate the Th17/Treg ratio in the spleen lymphocytes of AD mice in all dosage groups, H-M and H-H groups also significantly upregulated the Th1/Th2 ratio (Figures 6B, C). Based on the findings described, HLJDD potentially influences Th1/Th2 and Th17/Treg equilibrium through its modulation of OX40/OX40L expression.

### 3.7 Treatment of HLJDD altered the structure of intestinal and skin microbiota

**FIGURE 7:**
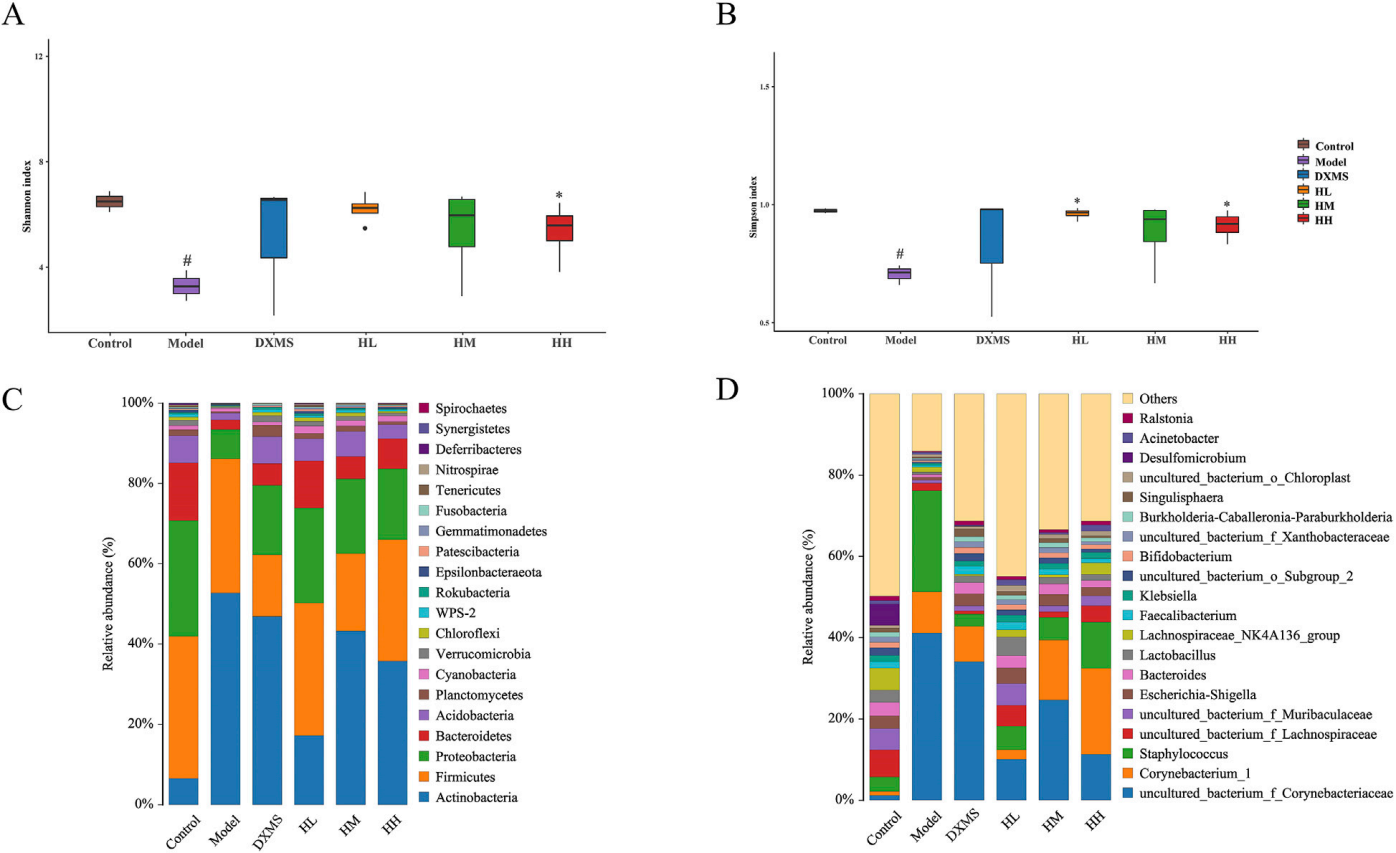
Treatment of HLJDD changed the skin microbial species diversity of AD-like mice **(A, B)** Alpha diversity analysis of each group was shown by Shannon and Simpson. The relative abundance of skin bacterial **(C)** phylum and **(D)** genus (n = 5). #*p* < 0.05 vs control group; *p < 0.05 vs model group.

In the model group, the diversity and structure of microbiota in the skin were decreased. Oral treatment of HLJDD could salvage these changes (Figures 7A, B). Alpha diversity analysis showed the imbalance of skin microbiota diversity in the model group, which was dominated by *uncultured_bacterium_f_Corynebacteriaceae*, *Corynebacterium_1*, and S*taphylococcus*. The relative abundance of Bacteroidetes, and *Acidobacteria* was reduced in the phylum level, which was increased by HLJDD and DXMS groups (Figure 7C). In the genus level, the relative abundance of *Staphylococcus* was increased in the model group, which was reduced by the treatment of HLJDD and DXMS (Figure 7D).

**FIGURE 8:**
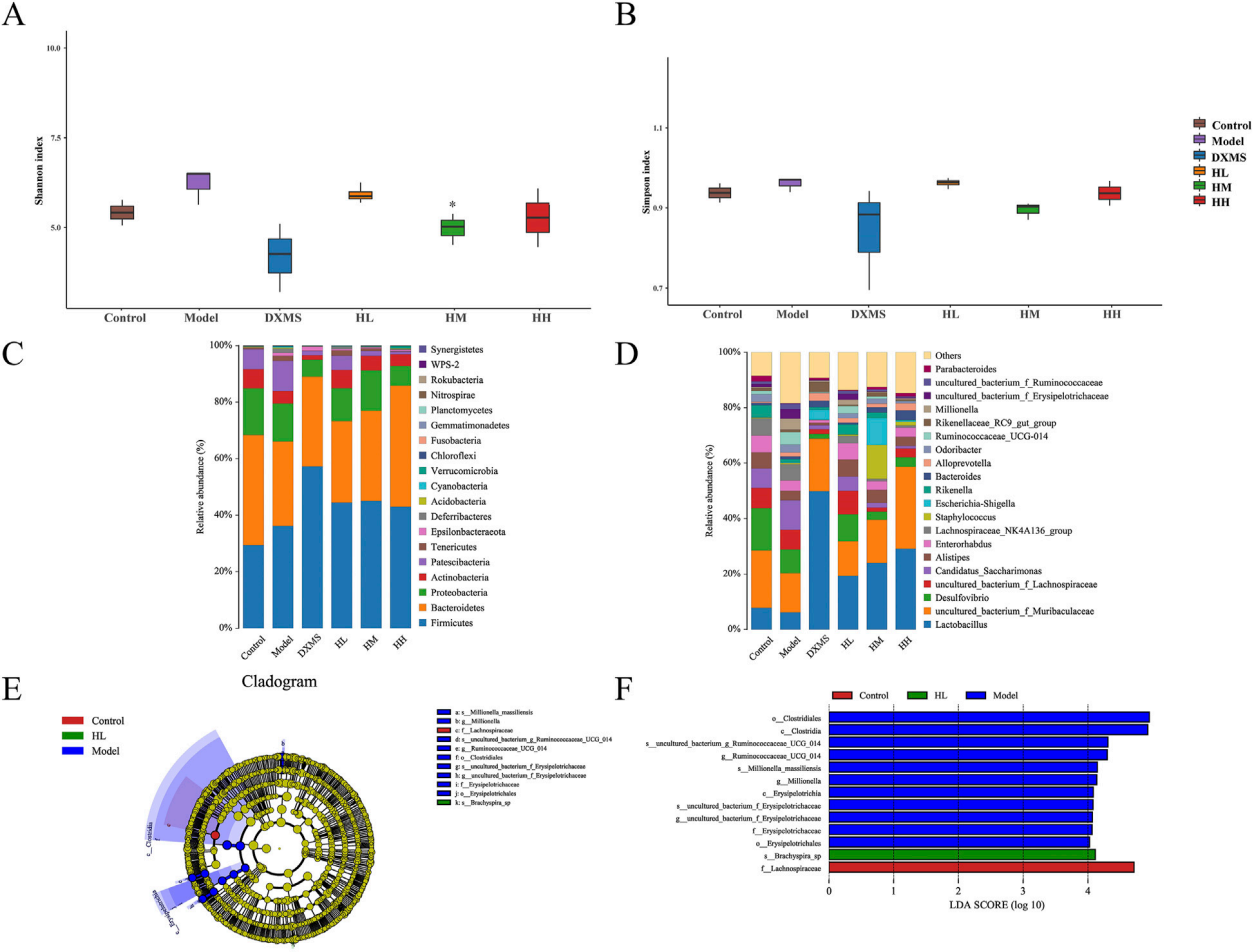
Effect of HLJDD changed the structure of fecal microbiota **(A, B)** Alpha diversity of each group was shown by Shannon and Simpson. The relative abundance of fecal bacterial **(C)** phylum and **(D)** genus (n = 5). **(E, F)** Analysis of differences in the microbial taxa shown by LEfSe upon Control group, HL group and Model group. *p < 0.05 vs model group.

As shown in Figures 8A, B, intestinal microbiota in the model group was slightly increased in diversity. In the phylum level, Actinobacteria in intestinal also showcased the opposite change compared with skin microbiota (Figures 7C, 8C).

In the genus level, *uncultured_bacterium_f_Muribaculaceae* and *Lactobacillus* were the prevailing microbial community in HLJDD and DXMS groups (Figure 8D). Species with significant differences were found in groups of control, model and high dose of HLJDD and most of the differential species belong to *Firmicutes* which were increased the relative abundance with treatment of HLJDD (Figures 8E, F).

### 3.8 Effect of HLJDD on the differentiation of naive CD4^+^ T cells *in vitro*

**FIGURE 9:**
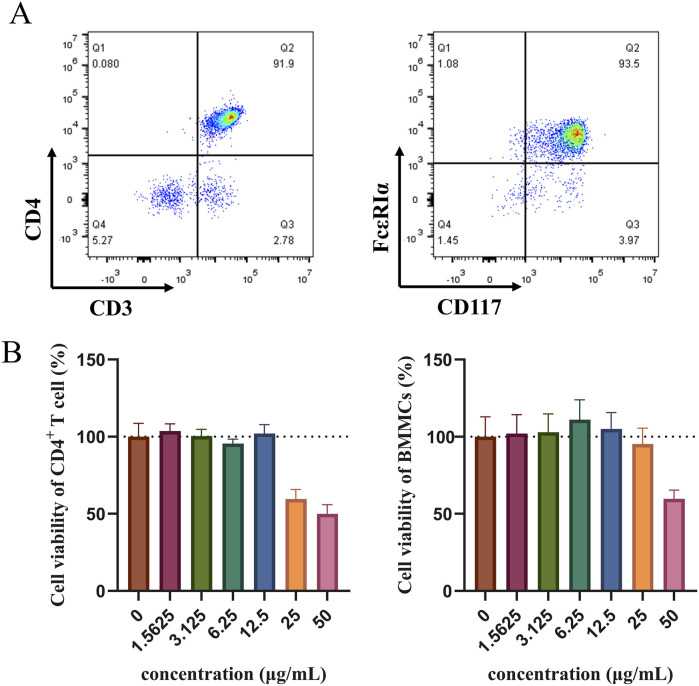
Detection of naive CD4^+^ T cells and BMMCs purity using flow cytometry and cell viability measured by CCK-8 **(A)** The purity results of naive CD4^+^ T cells and BMMCs were identified by flow cytometry *in vitro* experiments. **(B)** Effect of HLJDD on the viability of naive CD4^+^ T cells and BMMCs (n = 3). Data were the mean ± SEM.

**FIGURE 10:**
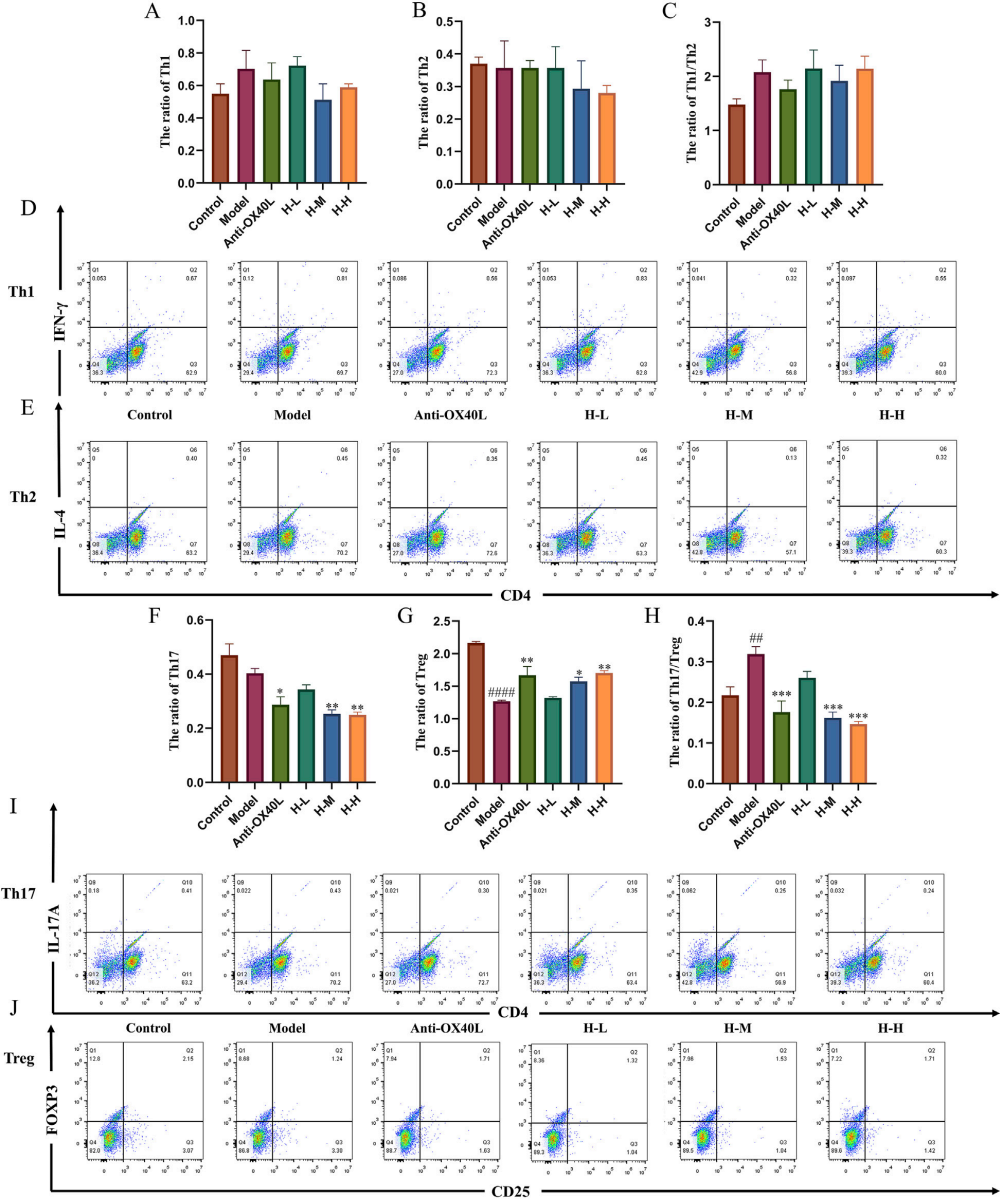
Effect of HLJDD on the differentiation of Th1/Th2 and Th17/Treg *in vitro* **(A–C)** Results of Th1, Th2, and Th1/Th2 ratios in each group (n = 3) **(D, E)** Representative images of Th1/Th2 detected by flow cytometry. **(F–H)** Results of Th17, Treg, and Th17/Treg ratios in each group (n = 3). **(I, J)** Representative images of Th17/Treg detected by flow cytometry. Data were the mean ± SEM. ##*p* < 0.01, ####*p* < 0.0001 vs control group; *p < 0.05, **p < 0.01, and ***p < 0.001 vs model group.

To verify the effect of the HLJDD on Th-cell differentiation by the OX40/OX40L signaling pathway, we extracted primary cells for validation and added OX40L blocker as a positive control. The purity identification results by flow cytometry analysis showed that the BMMCs and naive CD4^+^ T cells from the spleen had a purity of over 90% and could be used for subsequent research (Figure 9A). According to the CCK-8 results, 3.125, 6.25, and 12.5 μg/mL of HLJDD were used in the subsequent experiments (Figure 9B). Following IgE antigen sensitization and stimulation, no significant change was observed in the differentiation degree of Th1 and Th2 in each group when BMMCs and naive CD4^+^ T cells were co-cultured. Similarly, no significant change was noted in the Th1/Th2 ratio. (Figures 10A–C). However, compared with the control group, the ratio of Tregs in the model group was significantly reduced and the Th17/Treg ratio was significantly upregulated (Figures 10G, H). After treatment with a high-dose HLJDD, dramatically increased in the ratio of Treg, and decreased Th17 and Th17/Treg ratios were observed, which were similar to the Anti-OX40L group (Figures 10F–H). These results indicated that both intervention with HLJDD and blockade of OX40-OX40L signalling could downregulate the Th17/Treg ratio rather than the Th1/Th2 ratio.

## 4 Discussion

The main shield between the body and the environment is the skin barrier, which functions to prevent dryness and the entry of harmful allergens (Uberoi et al., 2021). When the skin barrier function is damaged, it becomes dry, resulting in itchy, flaky skin (Tominaga and Takamori, 2022).

Pruritus is a common symptom in AD patients (Labib et al., 2023). Scratching caused by itching could further damage the skin barrier, allowing uncontrolled entry of toxins and allergens into the body and exacerbating the inflammation in the skin. However, scratching could subsequently deteriorate the symptoms of AD and itch, eventually forming the itch-scratch cycle (Ständer, 2019). A damaged skin barrier is associated with the downregulation of barrier-related proteins such as LOR, AQPs, and filaggrin (FLG), involucrin (IVL) (Furue, 2020; Tricarico et al., 2022). AQP3 expressed in keratin-forming cells from the basal to the granular layers of the epidermis plays a key role in water retention (Tricarico et al., 2022). Significantly increased expression of AQP3 protein has commonly been observed in AD patients with damaged skin (Nakahigashi et al., 2011). The permeability of the water molecules can be evaluated by detecting TEWL. The results showed that TEWL increased with the upregulated AQP3 expression in AD-like mice, which is compatible with the preceding findings (Lu et al., 2023). The expression of FLG protein was restored in AD-like mice treated with HLJDD. TEWL was also ameliorated by the treatment of the HLJDD. After treatment with HLJDD, results showed that the skin lesion symptoms of AD-like mice were significantly ameliorated, and the low dose of HLJDD upregulated the expression of LOR, whereas there was no substantial increase in the expression of LOR in the medium- and high-dose HLJDD.

The itch-scratch cycle also interferes with life activities, so relieving symptoms of itching could have a positive effect on the standard of living in AD patients (Garcovich et al., 2021). Inflammatory cells are attracted by scratches to infiltrate into the skin. In the present study, the epidermal mast cells and thickening of the epidermal were significantly reduced in the medium- and high-dose HLJDD. The scratching behavior in mice was significantly decreased after treatment of HLJDD. Apart from damaged skin barrier results from the itch-scratch cycle, pruritus from AD is based on signaling between pruritogens released by mast cells, T cells, eosinophils and keratinocytes, and immune cells (Ständer, 2021). HLJDD not only could repair the skin barrier but also relieved the symptoms of itching in AD-like mice. TSLP, IL-4, IL-13 and IL-31 are both mighty pruritogenic cytokines and pro-inflammatory mediators (Cevikbas et al., 2014; Wilson et al., 2013). Histamine is released from MCs as a classical itch factor in response to allergenic stimulation (Shim and Oh, 2008). It could induce an itch sensation by interacting with histamine receptors. HRH4 is associated with MCs and T cells activation, promotes the response of Th2 immunity, which results in the production of IL-31 (Gutzmer et al., 2009). It is well known that HRH4 antagonists could improve skin inflammation and pruritus by decreasing chemokines (Cowden et al., 2010). After HLJDD treatment, the increased scratching behaviors in AD-like mice were associated with reduced mast cell infiltration, along with a distinct downregulation of TSLP, IL-13, IL-31, and HRH4 mRNA.

A key feature of AD skin lesions is an infiltration consisting primarily of T cells. Specifically, in serum, Th2-type biomarkers showcased a significant positive correlation with AD severity. Patients with moderate-to-severe AD displayed biomarkers dominated by Th2 profiles (Wu et al., 2023). Thus, the dysregulation ratio of CD4^+^ T cell subsets represents a crucial role in the pathophysiology of AD (Eapen et al., 2022). It is established that infiltrating Th2 cells in AD skin lesions release IL-4 and IL-13, which promote the production of antigen-specific IgE by B cells. IgE receptor in MCs binds to the IgE, which leads to MCs degranulation (Zheng et al., 2023). An imbalance between Th17 and Treg cells has been observed in both skin lesions and blood of AD patients (Ma et al., 2014). Our experiments showed that AD-like mice had significantly higher Th1/Th2 ratios and lower Th17/Treg ratios, showing a trend of Th2 and Th17 drift, which was consistent with the immune characteristics of the acute phase of AD. After treatment with medium- and high-dose HLJDD, the trend of Th-cell differentiation was ameliorated in AD-like mice.

Damaged skin barrier in AD shows an upregulation level of antigen-presenting cells (APCs) that uptake and process IgE-bound allergens and participate in T-cell stimulation (Novak et al., 2004). OX40 plays a pivotal role in the proliferation and survival of T cells by serving as a crucial co-stimulatory molecule. OX40L is located in APCs, including MCs (Webb et al., 2016). BMMCs present the antigen to CD4^+^ T cell to promote the activation and proliferation of CD4^+^ T cells through OX40/OX40L cell-cell interactions (Bulfone-Paus and Bahri, 2015). Th1, Th2, Th17, and Treg-mediated immune responses are associated with OX40/OX40L, all of which have been concerned in AD. OX40/OX40L signal has the potential to enhance Th1-mediated immune response, promote the generation and continuity of Th2-related immune response, and prevent Treg production and Treg-mediated suppression of immunity (Fu et al., 2020). Moreover, the expression of OX40 and OX40L is increased in AD (Croft et al., 2024). The suppressed Tregs and enhanced Th-cells related immune response could bring about the increasing production of pro-inflammatory factors. This phenomenon would exacerbate the itch-scratch cycle, thereby further impairing the integrity of the skin barrier. In this study, we found that OX40/OX40L expression was upregulated in the lesional skin of AD-like mice and was accompanied by Th2 and Th17 dominant phenotyping. The results of *in vitro* experiments also reflected Th17-dominant Th-cell phenotyping. Based on the above findings, the role of HLJDD in regulating Th1/Th2, Th17/Treg homeostasis in AD mice is likely to be related to its regulation of OX40 and OX40L expression.

*Firmicutes* and *Bacteroidetes* are associated with producing short-chain fatty acids (SCFAs) (Hu et al., 2020). Evidence showed that Tregs function and the generation of Tregs in human skin are enhanced by SCFAs (Schwarz et al., 2017; Xiao et al., 2022). Skin in AD patients is often colonized by *Staphylococcus aureus*, which is correlated with AD severity (Focken et al., 2023). As symptoms were alleviated, HLJDD groups could enhance the skin microbiota diversity and reduce the colonization of *Staphylococcus*. Our results showed that Th cells-related immune response and skin barrier function were alleviated by HLJDD, which may offer a stable environment for skin microbiota, and thus, the diversity of skin microbiota was enhanced. In fecal samples, both saw an increased relative abundance of *Firmicutes* and *Bacteroidetes* in the HLJDD groups. Probiotic *Lactobacillus* also showcased a positive effect in treating AD (Xie et al., 2023). The results indicated that HLJDD may enhance the abundance of SCFAs-producing bacteria and probiotics to alleviate the intestinal microbiota disorder. This suggested that HLJDD had influence on commensal microbiota.

HLJDD has been employed in clinical practices for the treatment of AD (Liu, 2010; Suqing and Yuepeng, 2017). The clinical utilisation of HLJDD could decrease corticosteroids use and assist in curbing the reliance on corticosteroids and alleviate symptoms of steroid-induced dermatitis (Tao, 2023). In addition, 3-week treatment with HLJDD in conjunction with anti-histamine drugs could improve skin conditions and did not show acute toxicity in children with AD (Wenzheng Zhu et al., 2023). However, it has been reported that some herbs or metabolites in HLJDD may have hepatotoxicity and nephrotoxicity (Tian et al., 2018; Wang et al., 2024). Therefore, randomized, double-blind, placebo-controlled clinical trials of HLJDD for AD treatment are necessary for evaluating its efficacy and long-term safety.

Our findings demonstrated that HLJDD was effective in disrupting the itch-scratch cycle and influencing the differentiation of Th cells in AD-like mice, and also indicated that scratching behaviors and pruritus-related cytokines were reduced in AD-like mice. Nevertheless, both human skin and the gut microbiota exert an influence on AD (Park et al., 2021). Our results showed that HLJDD exerts disparate effects on the skin and gut microbiota in AD-like mice. This finding suggests that the therapeutic efficacy of HLJDD in treating AD may be mediated through the gut-skin axis, thereby enhancing skin barrier function. It has been reported baicalin from HLJDD could regulate the gut microbiota of mice and restore the skin barrier function in AD-like mice (Wang et al., 2022). Research has demonstrated that geniposide and berberine, two metabolites of HLJDD, possess the ability to mitigate oxidative stress, a significant pathological factor to the development of AD (Bertino et al., 2020; Cao et al., 2021; Zou et al., 2020). However, our work still has limitations, the specific mechanism and active metabolites of HLJDD in regulating the OX40/OX40L signal and gut-skin axis are still not clear, which are still worth of further investigation.

## 5 Conclusion

**FIGURE 11:**
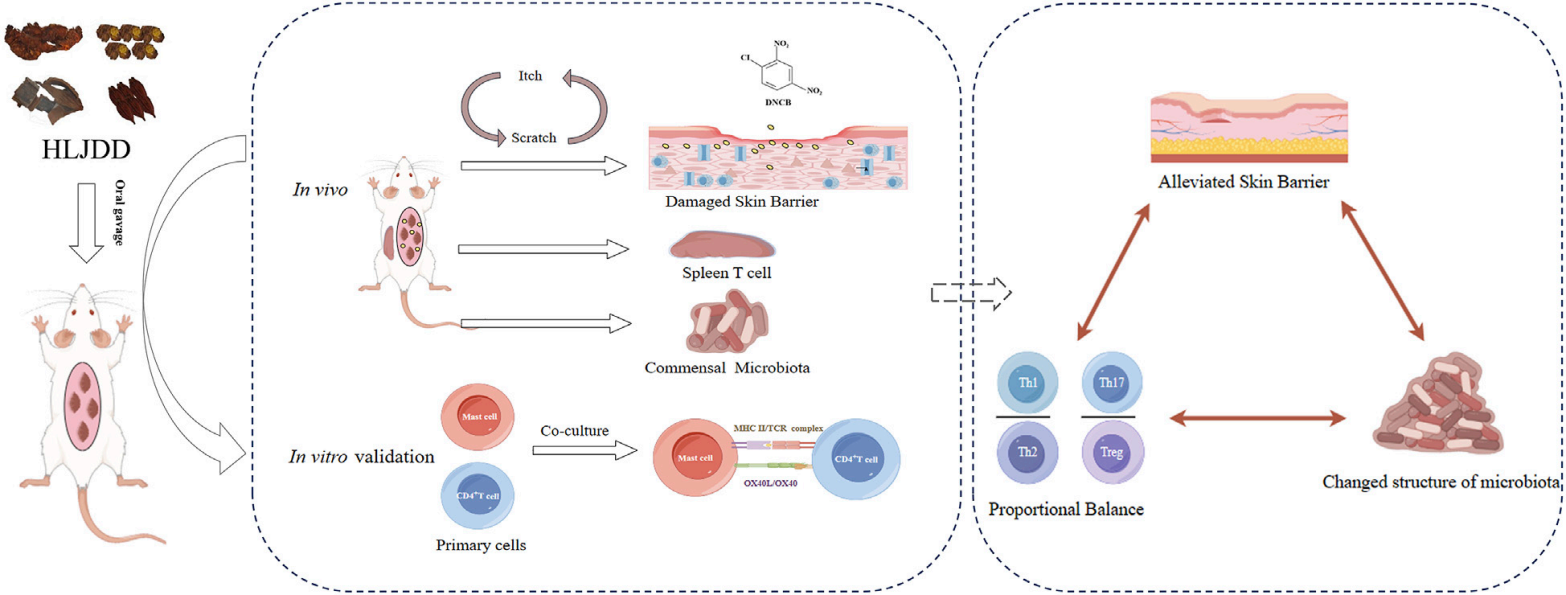
HLJDD alleviates symptoms in AD-like mice by regulating T helper cell differentiation through modulating OX40/OX40L signaling pathway, improves skin barrier function and influences commensal microbiota. The materials presented in this figure were partially sourced from Figdraw.

Generally, our experimental findings demonstrated that HLJDD could ameliorate skin symptoms and change the relative diversity of microbiota in AD-like mice (Figure 11). Briefly, HLJDD could break the itch-scratch cycle by reducing the IL-13, IL-31, TSLP, HRH4 mRNA and MCs infiltration. Furthermore, HLJDD elevated the expression of FLG and LOR while reducing the expression of AQP3 in the skin, which consequently augmented the skin barrier function. It was attributed to the HLJDD’s effect of balancing the Th1/Th2, Th17/Treg ratios via decreased expression of OX40/OX40L. *In vitro* experiment results showed that HLJDD could regulate Th17/Treg balance, which demonstrates the regulatory role of HLJDD in immunity.

## Data Availability

The original contributions presented in the study are publicly available. The RNA sequencing data can be found via the NCBI repository (SRA), accession number PRJNA1185638. This is available at: https://www.ncbi.nlm.nih.gov/bioproject/PRJNA1185638.
